# Impact of changes at the *Candida albicans* cell surface upon immunogenicity and colonisation in the gastrointestinal tract^☆^

**DOI:** 10.1016/j.tcsw.2022.100084

**Published:** 2022-10-17

**Authors:** Gabriela M. Avelar, Ivy M. Dambuza, Liviana Ricci, Raif Yuecel, Kevin Mackenzie, Delma S. Childers, Judith M. Bain, Arnab Pradhan, Daniel E. Larcombe, Mihai G. Netea, Lars P. Erwig, Gordon D. Brown, Sylvia H. Duncan, Neil A.R. Gow, Alan W. Walker, Alistair J.P. Brown

**Affiliations:** a Aberdeen Fungal Group, University of Aberdeen, Institute of Medical Sciences, Foresterhill, Aberdeen AB25 2ZD, UK; b Medical Research Council Centre for Medical Mycology, University of Exeter, Geoffrey Pope Building, Stocker Road, Exeter EX4 4QD, UK; c Microbiome, Food Innovation and Food Security Research Theme, Rowett Institute, University of Aberdeen, Foresterhill, Aberdeen AB25 2ZD, UK; d Iain Fraser Cytometry Centre, Institute of Medical Sciences, Foresterhill, Aberdeen AB25 2ZD, UK; e Microscopy & Histology Facility, Institute of Medical Sciences, Foresterhill, Aberdeen AB25 2ZD, UK; f Department of Internal Medicine and Radboud Center for Infectious Diseases, Radboud University Medical Center, Nijmegen, Netherlands; g Department for Immunology & Metabolism, Life and Medical Sciences Institute (LIMES), University of Bonn, 53115 Bonn, Germany; h Johnson-Johnson Innovation, EMEA Innovation Centre, One Chapel Place, London W1G 0BG, UK

**Keywords:** *Candida albicans*, Gut colonisation, β-Glucan exposure, Cell wall, Fungal immunogenicity

## Abstract

The immunogenicity of *Candida albicans* cells is influenced by changes in the exposure of microbe-associated molecular patterns (MAMPs) on the fungal cell surface. Previously, the degree of exposure on the *C. albicans* cell surface of the immunoinflammatory MAMP β-(1,3)-glucan was shown to correlate inversely with colonisation levels in the gastrointestinal (GI) tract. This is important because life-threatening systemic candidiasis in critically ill patients often arises from translocation of *C. albicans* strains present in the patient’s GI tract. Therefore, using a murine model, we have examined the impact of gut-related factors upon β-glucan exposure and colonisation levels in the GI tract.

The degree of β-glucan exposure was examined by imaging flow cytometry of *C. albicans* cells taken directly from GI compartments, and compared with colonisation levels. Fungal β-glucan exposure was lower in the cecum than the small intestine, and fungal burdens were correspondingly higher in the cecum. This inverse correlation did not hold for the large intestine.

The gut fermentation acid, lactate, triggers β-glucan masking *in vitro,* leading to attenuated anti-*Candida* immune responses. Additional fermentation acids are present in the GI tract, including acetate, propionate, and butyrate. We show that these acids also influence β-glucan exposure on *C. albicans* cells *in vitro* and, like lactate, they influence β-glucan exposure via Gpr1/Gpa2-mediated signalling. Significantly, *C. albicans gpr*1Δ *gpa*2Δ cells displayed elevated β-glucan exposure in the large intestine and a corresponding decrease in fungal burden, consistent with the idea that Gpr1/Gpa2-mediated β-glucan masking influences colonisation of this GI compartment. Finally, extracts from the murine gut and culture supernatants from the mannan grazing gut anaerobe *Bacteroides thetaiotaomicron* promote β-glucan exposure at the *C. albicans* cell surface. Therefore, the local microbiota influences β-glucan exposure levels directly (via mannan grazing) and indirectly (via fermentation acids), whilst β-glucan masking appears to promote *C. albicans* colonisation of the murine large intestine.

## Graphical abstract

**Figure:**
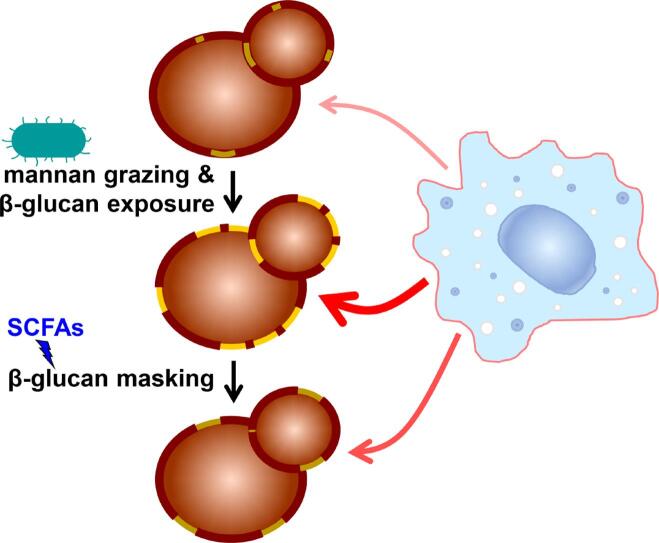

## Introduction

The opportunistic fungal pathogen *Candida albicans* exists as a commensal of the gastrointestinal tract and mucosae in many individuals, and is a frequent cause of mucosal and systemic infection (Brown et al., 2012, Kohler et al., 2014, Pappas et al., 2018, Yano et al., 2019, Hertel et al., 2016). Indeed, *C. albicans* is the leading cause of hospital-acquired fungal bloodstream infections (Pappas et al., 2018).

The gut mycobiota provides protective functions by inducing antifungal antibodies (Doron et al., 2021a, Doron et al., 2021b). Nevertheless, many systemic candidemia episodes in hospitalised patients originate from the gut (Miranda et al., 2009, Gouba and Drancourt, 2015, Zhai et al., 2020). The translocation of *C. albicans* cells from gut to bloodstream is enhanced by compromised immunity and neutropenia in particular, but also by impaired intestinal barrier function (caused by surgery, traumatic injury or conditions such as inflammatory bowel disease) and by fungal overgrowth in the gut (caused by antibiotic treatments that perturb the intestinal microbiota which normally provides colonisation resistance) (Koh et al., 2008, Lionakis, 2014, Desai and Lionakis, 2018, Mishra and Koh, 2018, Zhai et al., 2020). Despite the prevalence of *C. albicans* as a gut commensal, the factors that influence gastrointestinal colonisation remain poorly understood in comparison with those that affect systemic infection. Therefore, the significance of *C. albicans* as a gut commensal is receiving increased attention (Neville et al., 2015, Paterson et al., 2017, Mishra and Koh, 2018, Pérez, 2019, Kumamoto et al., 2020, Romo and Kumamoto, 2020).

Numerous factors influence the transition from gut commensalism to infection, which involves multivariate interactions between *C. albicans,* the host and the gut microbiota (Mishra and Koh, 2018, d'Enfert et al., 2021). Resident immune cells protect against *C. albicans* invasion and translocation (Leonardi et al., 2018), exploiting specific pattern recognition receptors (PRRs) to recognise cognate microbe-associated molecular patterns (MAMPs) and activate antifungal immune responses (Netea et al., 2008, Brown, 2011, Dambuza et al., 2017, Netea et al., 2017). Host-generated mucins and bile acids provide additional protection by suppressing *C. albicans* virulence traits (Kavanaugh et al., 2014, Guinan et al., 2019). Meanwhile, the gut microbiota limits the outgrowth of *C. albicans* via colonisation resistance, a phenomenon that includes the manipulation of gut pH and oxygen levels (Ubeda et al., 2010, Lawley and Walker, 2013, d'Enfert et al., 2021), as well as competition for nutrients and the generation of inhibitory metabolites such as short chain fatty acids (SCFAs) (Cummings, 1981, Kennedy and Volz, 1985a, Louis et al., 2007, Guinan et al., 2019, Ricci et al., 2021). For example, the SCFAs acetate, propionate and butyrate inhibit the growth and morphogenesis of *C. albicans in vitro* (Cottier et al., 2015, Cottier et al., 2017, Noverr and Huffnagle, 2004). Also, antibiotic treatments that compromise cecal bile acid concentrations and elevate fecal *C. albicans* burdens in rodents (Kennedy and Volz, 1985a, Kennedy and Volz, 1985b, Guinan et al., 2019) often permit gut colonisation (Fan et al., 2015).

The regulatory circuitry in *C. albicans* that promotes gut commensalism overlaps with that required for systemic infection (Perez et al., 2013). The overlaps between these regulatory circuits largely reflect the importance of metabolic adaptation for fungal fitness in the host, whereas the differences highlight the contrasting roles of yeast-hypha morphogenesis during gut commensalism and systemic infection (Perez et al., 2013). Hyphal development promotes systemic infection, but compromises gut commensalism (Saville et al., 2003, Witchley et al., 2019), and the hyphal form is the target for protective antifungal antibodies in the gut (Doron et al., 2021b). The master regulator Efg1 promotes hyphal development in *C. albicans.* Efg1 attenuates gut colonisation (Stoldt et al., 1997, Pierce et al., 2013), and this leads to the emergence of Efg1-attenuating mutations in the GI tract or gut-like environments (Ene et al., 2018, Tso et al., 2018, Liang et al., 2019). The inactivation of other factors associated with hyphal development (such as Ume6 and Flo8) or hypha-associated factors (like Sap6 and Hyr1) also enhances gut commensalism (Witchley et al., 2019, Tso et al., 2018). Wor1 acts in opposition to Efg1 within a complex transcriptional circuit that regulates *C. albicans* cell type and morphology (Zordan et al., 2007, Nobile et al., 2012, Park et al., mSphere 2020). Wor1 enhances gut colonisation (Pande et al. 2013), and ectopic *WOR1* expression promotes a gastrointestinally-induced transition (GUT) phenotype in *C. albicans* that displays increased fitness during gut commensalism (Pande et al., 2013, Noble et al., 2017). Therefore, while both yeast and hyphal morphologies have been observed in the gut (Witchley et al., 2019), the yeast form promotes persistence during gut commensalism (Böhm et al., 2017).

The yeast form of *C. albicans* is a moving target for the immune system because yeast cells alter the exposure of β-glucan on their cell surface in response to specific environmental inputs (Ballou et al., 2016, Sherrington et al., 2017, Pradhan et al., 2019, Pradhan et al., 2018, Cottier et al., 2019). β-glucan is a proinflammatory MAMP that, together with the cognate PRR dectin-1, plays key roles in antifungal immunity in mice and humans (Brown and Gordon, 2001, Brown et al., 2002, Taylor et al., 2007, Ferwerda et al., 2009, Plantinga et al., 2009, Carvalho et al., 2012). In *C. albicans*, most β-glucan is present in the inner layer of the cell wall, masked from immune recognition by an outer layer of mannan fibrils (Erwig and Gow, 2016, Graus et al., 2018, Lenardon et al., 2020). Phagocytic attack can lead to exposure of β-glucan at the cell surface via stripping of the mannan outer layer by immune cells (Wheeler et al., 2008). However, even in the absence of phagocytic attack, some β-glucan can become exposed in punctate features on the cell surface and at septal junctions and bud scars (Pradhan et al., 2019, Childers et al., 2020, de Assis et al., 2021). *C. albicans* cells modulate their degree of β-glucan exposure in response to host- and microbiota-related environmental signals, such as lactate, ambient pH, hypoxia and iron depletion (Ballou et al., 2016, Sherrington et al., 2017, Pradhan et al., 2019, Pradhan et al., 2018, Cottier et al., 2019), partly by re-masking exposed β-glucan and partly by shaving it from the cell surface (Childers et al., 2020, Cottier et al., 2019). This ability to modulate the exposure of an inflammatory MAMP at its cell surface significantly affects immune responses against *C. albicans in vitro* and *in vivo* (Ballou et al., 2016, Sherrington et al., 2017, Pradhan et al., 2018, Pradhan et al., 2019, Cottier et al., 2019, Lopes et al., 2018, de Assis et al., 2021).

Elevated β-glucan exposure has been observed during vaginal candidiasis (Pericolini et al., 2018), which resonates with the immunopathology often associated with this condition (Fidel et al., 2004, Yano et al., 2019, d'Enfert et al., 2021). Lactate, a fermentation acid present in the gut (Cummings, 1981, Louis et al., 2007), triggers β-glucan masking (Ballou et al., 2016). In principle, this would be expected to enhance colonisation. Consistent with this idea, analyses of fecal burdens for β-glucan exposing *C. albicans* cell wall mutants have suggested that β-glucan exposure compromises colonisation of the murine gut (Sem et al., 2016). However, the inactivation of the β-glucan receptor dectin-1 does not appear to enhance *C. albicans* colonisation of the gut (Vautier et al., 2012), suggesting that other receptors might play a role, or that β-glucan exposure might not influence colonisation levels. Therefore, using a mouse model, we set out to explore the levels of β-glucan exposure on *C. albicans* cells in different gastrointestinal compartments. Our data suggest that lactate-induced β-glucan masking does promote colonisation of certain gastrointestinal compartments in a Wor1-independent fashion. Nevertheless, consistent with the complexity of fungus*-*host-microbiota interactions in the gut (d'Enfert et al., 2021), additional factors influence gut colonisation by *C. albicans.*

## Materials and Methods

### Strains and growth conditions

*C. albicans* strains are listed in Supplementary Table 1. For all experiments, *C. albicans* strains were pre-grown overnight at 30 °C, 200 rpm in minimal medium (GYNB: 2 % glucose, 0.65 % yeast nitrogen base without amino acids, containing the appropriate supplements) (Sherman, 1991). On the day of an experiment, overnight cultures were diluted into fresh minimal medium to an OD_600_ of 0.2 and incubated at 30 °C at 200 rpm for 5 h for analysis. In some experiments, *C. albicans* cells were diluted into colon-simulating medium (CSM) containing xylan 0.6 g/L, pectin 0.6 g/L, amylopectin 0.6 g/l, arabinogalactan 0.6 g/l, potato starch 5 g/l, casein hydrolysate 3 g/l, peptone water 3 g/L, K_2_HPO_4_ 2 g/L, NaHCO_3_ 0.2 g/L, NaCl 4.5 g/L, MgSO_4_·7H_2_O 0.5 g/L, CaCl_2_·2H_2_O 0.45 g/L, FeSO_4_·7H_2_O 0.005 g/L, haemin 0.01 g/L, bile salts 0.05 g/L, Antifoam A 0.5 ml/L, resazurin (0.1 %) 0.6 ml/L, acetate 33 mM, propionate 9 mM, butyrate 5 mM, isobutyrate 1 mM, isovalerate 1 mM, valerate 1 mM, lactate 20 mM, EDTA 1 mg/L, FeSO_4_·7H_2_O 0.4 mg/L, ZnSO_4_·7H_2_O 0.02 mg/L, MnCl_2_·7H_2_O 0.006 mg/L, H_3_BO_3_ 0.06 mg/L, CoCl_2_·6H_2_O 0.04 mg/L, CuCl_2_·2H_2_O 0.002 mg/L, NiCl_2_·6H_2_O 0.004 mg/L, NaMoO_4_·2H_2_O 0.006 mg/l, menadione 1.4 mg/L, biotin 2.8 mg/L, pantothenate 2.8 mg/L, nicotinamide 14 mg/L, vitamin B_12_ 0.7 mg/L, thiamine 5.6 mg/L, p-aminobenzoic acid 7 mg/L, cysteine 20 mg/L, NaHCO_3_ 4.2 mg/L. This medium was adapted from Macfarlane *et al.* (Macfarlane et al., 1989).

*B. thetaiotaomicron* B5482 (DSM 2079) (Russell et al., 2013), *B. adolescentis* L2-32 (Barcenilla et al., 2000) and *C. eutactus* ART55/1 (Alessi et al., 2020) were obtained from strain collections at the Rowett Institute, University of Aberdeen, UK, and grown in anaerobic M2GSC medium (Miyazaki et al., 1997) at 37 °C overnight (Ricci et al., 2021). Note that *B. thetaiotaomicron* B5482 (DSM 2079) is the same strain as VPI-5482, which was used previously to examine mannan degradation (Cuskin et al., 2015).

### Microscopy

For fluorescence microscopy, *C. albicans* cells were fixed in 50 mM thimerosal and stained for exposed β-glucan (1.5 µg/ml Fc-Dectin-1 plus anti-human IgG conjugated to Alexafluor 488; green) and exposed mannan (25 µg/ml Concanavalin A conjugated to Alexafluor 647; red). All samples were examined by phase differential interference contrast (DIC) and fluorescence microscopy using a Zeiss Axioplan 2 microscope. Images were recorded digitally using Openlab v 4.04 (Improvision, Coventry, UK) with a Hamamatsu C4742- 95 digital camera (Hamamatsu Photonics, Japan). Fluorescence was quantified using and processed using Openlab (Openlab v 4.04: Improvision, Coventry, UK).

*C. albicans* cell walls were examined by high-pressure freeze substitution transmission electron microscopy (TEM). *C. albicans* SC5314 cells were incubated for 6 h at 37 °C with extracts from the small intestine, cecum or large intestine (above). These fungal cells were then processed for TEM as described previously (Ene et al., 2012), cutting ultrathin (100 nm) sections. Samples were imaged with a Philips CM10 transmission microscope (FEI, United Kingdom) equipped with a Gatan Bioscan 792 camera, and the images recorded using a Digital Micrograph (Gatan, Abingdon Oxon, United Kingdom).

### Flow cytometry

Levels of MAMP exposure by *C. albicans* cells were quantified by flow cytometry as described previously (Pradhan et al., 2019, Pradhan et al., 2018). Briefly, cells were grown on minimal medium, exposed to the specified inputs for 5 h, and fixed with 50 mM thimerosal. To examine β-glucan exposure, the cells were then stained with 1.5 µg/ml Fc-Dectin-1 and anti-human IgG conjugated to Alexafluor 488, and to monitor mannan exposure cells were then stained with 25 µg/ml Concanavalin A conjugated to Alexafluor 647. The fluorescence of 10,000 cells was acquired using a BD Fortessa flow cytometer and Median fluorescence intensities (MFIs) were determined using FlowJo software v10 (BD Fortessa flow cytometer). Cells from the small intestine, cecum and large intestine were analysed in the Amnis ImageStreamX MK II imaging flow cytometer (Luminex, Austin, TX, USA). Data were obtained from at least three independent biological replicates. Morphological features (cell size and circularity) were used to isolate yeast cells. Data analysis was performed using IDEAS software (version 6.2, Luminex, Austin, TX, USA). The gating strategy and axis scales, which remained unchanged throughout, are presented in Supplementary Fig. 2.

### qRT-PCR

Transcript levels were measured by qRT-PCR. Total mRNA was isolated from the contents of murine gut compartments (above) and from control *C. albicans* cells grown *in vitro* on GYNB (above) using TRIzol (Invitrogen, Paisley, UK) and FastPrep-24 (MP Biomedicals, Luton, UK) according to the manufacturer’s instructions. RNA preparations were treated with DNase (Turbo DNase; Ambion, Banchory, UK) and stored at −80 °C. For reverse transcription, Superscript II Reverse Transcriptase (Invitrogen) kits were used with 1 µg of total RNA as per the manufacturer’s instructions. qPCR was performed on these cDNA preparations using a Rotor Gene Q thermocycler (Qiagen) with gene-specific primers (Supplementary Table 2) and Takyon SYBR® Master Mix (Takyon No Rox SYBR® MasterMix dTTP Blue, UF-NSMT-B0701: Eurogentec, Seraing, Belgium). Fold changes in gene expression levels were derived using the ΔCt method (Edmunds et al., 2014) and the data were analysed statistically using one-way analysis of variance (ANOVA). The data were expressed relative to the internal *ACT1* mRNA control and then normalized against the transcript levels in control *C. albicans* cells grown *in vitro* (above).

### Fermentation acids analysis

Short chain fatty acids (SCFAs) and lactate were extracted from samples collected from gut compartments using diethyl ether and derivatised using *N*-*tert*-butyldimethylsilyl-*N*-methyltrifluoroacetamide, then quantified by analysing these extracts by gas–liquid chromatography using a Hewlett Packard gas chromatograph (GC) fitted with a silica capillary column using helium as the carrier gas as described previously (Richardson et al., 1989). For each experiment, 2-ethyl butyrate as used as an internal standard and an SCFA mixture as an external standard. SCFA concentrations were determined relative to standards of known SCFA concentration, and relative to the internal standard.

### Cytokine assays

Cytokine assays were performed using published procedures (Pradhan et al., 2018, Ballou et al., 2016). Briefly, peripheral blood mononuclear cells (PBMCs) were prepared from non-heparinised whole blood (20 ml) collected from healthy volunteers by Ficoll-Paque centrifugation according to the manufacturer’s instructions (Sigma-Aldrich) using EDTA as an anticoagulant (Greiner). Thimerosal-fixed *C. albicans* cells were washed with sterile PBS four times, mixed with PBMCs (5 yeast to 1 PBMC) and samples incubated for 24 h at 37 °C. Supernatants were then collected, and TNF-α, IL-6 and IL-10 quantified as per the manufacturer’s instructions using Luminex® Screening kits (R&D Systems, Abingdon, UK) in the BioPlex 200 System (Bio-Rad, Watford, UK). Each data point represents one sample of two from four different individuals.

### Murine model of gastrointestinal colonisation

The murine gastrointestinal model was used essentially as described previously (Vautier et al., 2012). Eight- to twelve-week-old female C57BL/6 mice were bred and maintained in the Biological Services Unit at Foresterhill, University of Aberdeen. The mice were housed separately in individually ventilated cages and provided with food and water *ad libitum*. To reduce the endogenous gut microbiota and mycobiota, mice were provided with sterile drinking water containing 2 mg/ml streptomycin, 2,000 U/ml penicillin, 0.25 mg/ml fluconazole (Enzo) for three days and then switched to water containing streptomycin and penicillin for a further 24 h before exposure to *C. albicans*. To prepare the *C. albicans* cells, strains were grown overnight in minimal medium, diluted into fresh minimal medium and regrown for 5 h, as described above. The *C. albicans* cells were washed twice in sterile phosphate-buffered saline (PBS), counted using a hemocytometer, the cell densities adjusted with PBS, and these densities confirmed by plating (CFUs). Mice were gavaged with 1x10^7^ *C. albicans* cells, and the mice were maintained on sterile water containing 2 mg/ml streptomycin, 2,000 U/ml penicillin and 0.2 mg/ml gentamicin. Mice were sacrificed after four days of exposure to *C. albicans*, and the small intestine, cecum and large intestine harvested and washed three times with 1 ml sterile PBS. Tissues were weighed and then homogenized and split into samples that were used to assay fungal burdens (CFUs), SCFA concentrations, fungal β-glucan exposure (by cytometry) and transcript levels (by qRT-PCR). Each mouse experiment was repeated at least twice with three mice per experiment.

To measure fungal colonisation, tissue samples were serially diluted tenfold, 25 μl of each dilution plated on YPD (Sherman, 1991) containing 0.01 mg/ml vancomycin and 0.1 mg/ml gentamicin, and the plates grown overnight at 37 °C. Fungal burdens were expressed as CFU per g tissue weight. Statistical analyses were performed using the Mann-Whitney *U* test using Prism 5: * p ≤ 0.05; ** p ≤ 0.01; *** p ≤ 0.001.

### Gut extracts and culture supernatants from gut anaerobes

Extracts were prepared from the contents of gut compartments from antibiotic treated mice colonised with *C. albicans* SC5314. Mice were sacrificed after four days of colonisation, and their small intestine, cecum and large intestine harvested and washed three times with 1 ml sterile PBS. The tissues plus their contents were then weighed and homogenized in 2 ml PBS. The homogenates were then centrifugated at 15000 rpm for 10 min and the supernatants taken for analysis (below).

To generate culture supernatants from *B. thetaiotaomicron* B5482 (DSM 2079), *B. adolescentis* L2-32 and *C. eutactus* ART55/1, each organism was cultured in anaerobic Hungate tubes containing M2GSC medium with 30 % clarified bovine rumen fluid and incubated at 37 °C overnight (Miyazaki et al., 1997, Ricci et al., 2021). Cultures were then centrifuged at 658 × *g* for 10 min and the supernatants sterilised by passage through 0.2 μm syringe-driven filter units (Millex, Merck Millipore ltd, Cork, Ireland) (Ricci et al., 2021).

### Ethical statement

All relevant ethical regulations for work with human participants/samples were adhered to in this study. Blood was collected from healthy volunteers following their informed consent according to local guidelines and regulations approved by the College Ethics Review Board, University of Aberdeen (CERB/2012/11/676). Animal experiments were approved by University of Aberdeen Animal Welfare and Ethical Review Body. C57BL/6 mice were bred in-house and housed under specific pathogen-free conditions and were selected at random. No surgical procedures were performed on animals prior to humane culling by cervical dislocation.

## Results

### β-Glucan exposure on *C. albicans* cells differs between gut compartments in mice

**Fig. 1:**
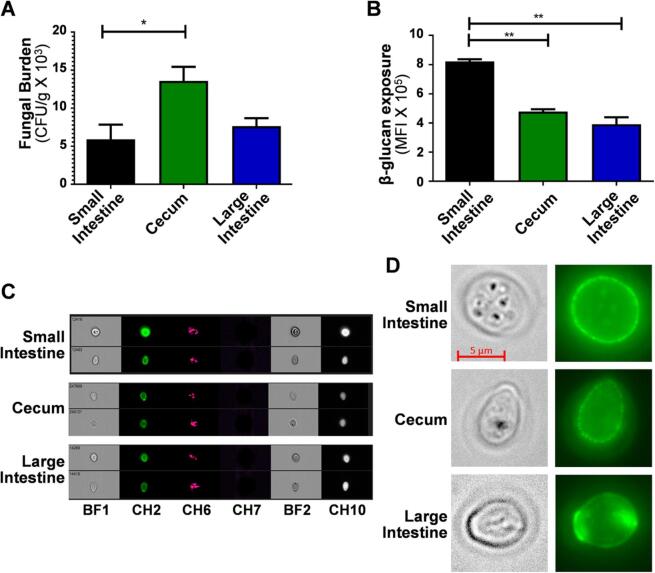
*C. albicans* cells isolated from murine gut compartments display different levels of β-glucan exposure. Mice were pre-treated with antibiotics and then colonised with *C. albicans* SC5314 by oral gavage. After four days the mice were sacrificed, and the contents of their small intestine, cecum and large intestine taken for analysis. (A) Fungal burdens were quantified by measuring CFUs per gram of tissue. (B) The β-glucan exposure of *C. albicans* cells from each gut compartment was determined by imaging flow cytometry after staining with Fc-dectin-1 and anti-human IgG conjugated to Alexafluor 488. Median fluorescence intensities (MFIs) for each *C. albicans* population are presented: means and standard deviations for n = 3 mice. Statistical analyses were performed using the Mann-Whitney *U* test using GraphPad Prism 5: * p ≤ 0.05; ** p ≤ 0.01. (C) Two events (from n = 10,000) from the imaging flow cytometry are shown for each gut compartment: BF1, bright field 1; CH2, AF488 (β-glucan exposure); CH6, side scatter; CH7, Hoechst 33,342 (DNA); BF2, bright field 2; CH10, ConA-AF647 (mannan exposure). (D) Magnifications of the DIC and fluorescence images for representative *C. albicans* cells from each gut compartment are presented.

A comparison of *C. albicans* cell wall mutants suggested an association between β-glucan exposure and reduced fitness during gut colonisation (Sem et al., 2016). Therefore, we compared *C. albicans* colonisation and β-glucan exposure levels directly for cells colonising different gastrointestinal compartments. To achieve this, mice were pre-treated with antibiotics to reduce the endogenous gut microbiota and mycobiota, and then colonised with the *C. albicans* clinical isolate SC5314. Fungal burdens (colony forming units: CFUs) were measured in the small intestine, cecum and large intestine after four days of colonisation because fungal colonisation has stabilised by this time in this model (Vautier et al., 2012). In parallel, the contents of each compartment were fixed, stained with Fc-dectin-1, and analysed by imaging flow cytometry. This approach permitted direct quantification of β-glucan exposure levels on *C. albicans* yeast cells in these gut compartments, selected on the basis of their size and form, but precluded the analysis of hyphal cells. Compared to the small intestine, fungal burdens in the cecum were approximately twofold higher (Fig. 1A), and β-glucan exposure levels on *C. albicans* cells were twofold lower (Fig. 1B–D). However, the fungal burdens were similar in the small and large intestine, although β-glucan exposure levels were significantly lower in the large intestine (Fig. 1A–D). Therefore, no obvious inverse correlation was observed between the levels of β-glucan exposure and *C. albicans* colonisation in these different gut compartments. Nevertheless, the data indicate that β-glucan exposure levels change, and hence that cell surface remodelling does occur, as *C. albicans* populations transit through the gut.

### Gut bacterial fermentation acids and β-glucan masking in gut compartments

**Fig. 2:**
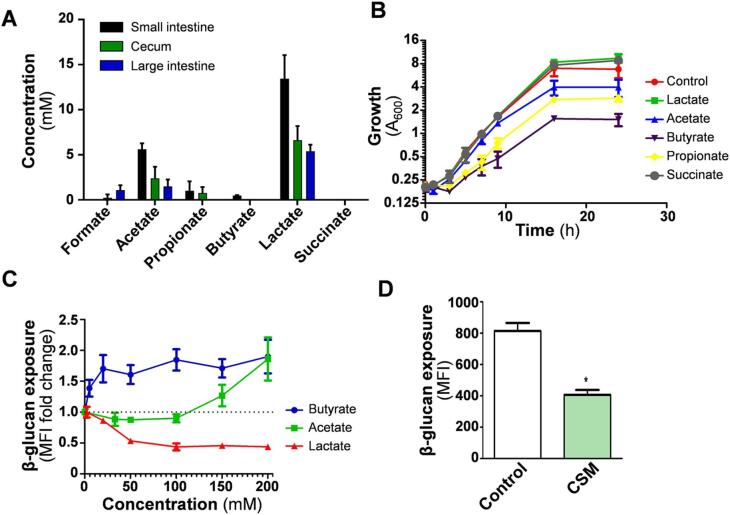
Bacterial fermentation acids in murine gut compartments and their impact on β-glucan exposure by *C. albicans*. (A) Fermentation acid concentrations were measured in the small intestine, cecum and large intestine of mice (n = 3) colonised for four days with *C. albicans* SC3154. (B) Impact of lactate (55 mM), acetate (83 mM), butyrate (56 mM), propionate (67 mM) and succinate (43 mM) upon the growth of *C. albicans* in GYNB at 30 °C. (C) Dose-dependent effects of lactate, acetate and butyrate upon β-glucan exposure levels for *C. albicans* SC5314 during growth *in vitro* in in GYNB at 30 °C. Cells were stained with Fc-dectin-1 (Fig. 1), their MFIs measured using a BD Fortessa flow cytometer, and β-glucan exposure levels expressed relative to the controls lacking SCFA. (D) The impact of colon simulating medium (CSM) on *C. albicans* β-glucan exposure, compared to control cells growing in GYNB. For all experiments, means and standard deviations are from three independent experiments (or n = 3 mice), and statistical analyses were performed using the Mann-Whitney *U* test using Prism 5: * p ≤ 0.05.

Changes in gut SCFA concentrations are thought to affect *C. albicans* colonisation in mice and humans (Mishra and Koh, 2018, Guinan et al., 2019, Gutierrez et al., 2020), potentially through their attenuation of *C. albicans* growth and morphogenesis (Noverr and Huffnagle, 2004, Nguyen et al., 2011, Cottier et al., 2015, Cottier et al., 2017, Guinan et al., 2019). Therefore, we measured bacterial fermentation acid levels in the luminal contents of the small intestine, cecum and large intestine of antibiotic pre-treated mice colonised by *C. albicans.* Overall, lactate was the most prevalent acid, followed by acetate, propionate, formate and butyrate, while succinate was not present at detectable concentrations (Fig. 2A). With the exception of formate, the concentrations of these acids were highest in the small intestine and lowest in the large intestine. The degree of β-glucan exposure on *C. albicans* cells in these same compartments was quantified by imaging flow cytometry (Fig. 1B). Lactate concentrations were about twofold higher in the small intestine compared to the large intestine (Fig. 2A), but β-glucan exposure was highest on the fungal cells in the small intestine (Fig. 1B). Therefore, the differences in β-glucan exposure observed between these gut compartments is not simply driven by lactate-induced β-glucan masking.

Factors other than lactate must be influencing β-glucan exposure within gut compartments. Therefore, we tested the effects of bacterial fermentation acids upon the growth and β-glucan exposure of *C. albicans* SC5314 cells *in vitro* on minimal medium, i.e. under conditions used previously to examine β-glucan masking (Ballou et al., 2016, Pradhan et al., 2018). Acetate and butyrate inhibited growth slightly under these conditions, whilst lactate exerted minimal effects upon growth (Fig. 2B). We then examined the effects of lactate, acetate and butyrate upon β-glucan exposure *in vitro* (Fig. 2C). As reported previously (Ballou et al., 2016), lactate induced β-glucan masking on *C. albicans* cells at physiologically relevant levels. When present at relatively low concentrations, butyrate enhanced β-glucan exposure in *C. albicans*, whereas acetate only promoted β-glucan exposure at high, non-physiological concentrations (Fig. 2C). We also tested normoxic growth in a colon-simulating culture medium, which included lactate (20 mM), acetate (33 mM) and butyrate (5 mM) as well as carbon sources mimicking those derived from commonly consumed dietary fibres, vitamins, micronutrients and salts (see Materials & Methods). β-glucan exposure was twofold lower after growth in colon simulating medium than the GYNB control condition (Fig. 2D), which was consistent with the relatively low levels of β-glucan exposure observed in the large intestine (colon) (Fig. 1B). Meanwhile, factors other than β-glucan exposure must be determining levels of fungal colonisation in the small intestine and cecum.

### Gpr1/Gpa2 signalling promotes β-glucan masking and *C. Albicans* colonisation in the large intestine

**Fig. 3:**
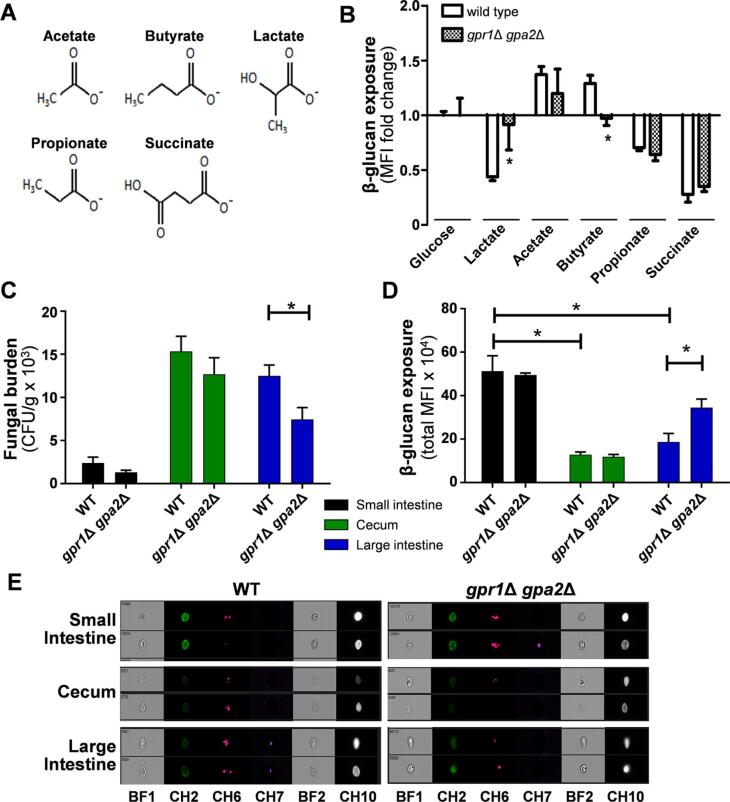
Impact of Gpr1/Gpa2 signalling on SCFA-mediated changes to *C. albicans* β-glucan exposure *in vitro* and *in vivo*. (A) Structural relationships between gut SCFAs. (B) The effects of SCFAs upon β-glucan exposure in *C. albicans* wild type control cells (Ca372) and the *gpr*1Δ *gpa*2Δ double mutant (NM23). *C. albicans* were grown *in vitro* in GYNB at 30 °C and transferred to fresh GYNB either lacking or containing the SCFA for five hours. The cells were then stained with Fc-dectin-1 (Fig. 1), their MFIs measured using a BD Fortessa flow cytometer, and β-glucan exposure levels expressed relative to the control lacking SCFA: 55 mM lactate, 83 mM acetate, 56 mM butyrate, 67 mM propionate, and 43 mM succinate. (C) Mice (n = 3) were colonised with *C. albicans* wild type cells (Ca372) or the *gpr*1Δ *gpa*2Δ double mutant (NM23). After four days the mice were sacrificed, and fungal burdens quantified (CFUs) in the small intestine, cecum and large intestine. (D) The levels of β-glucan exposure on *C. albicans* cells from the same gut compartments were measured by Fc-dectin-1 staining and imaging flow cytometry (Fig. 1). (E) Two events (from n = 10,000) from the imaging flow cytometry in Fig. 3D are shown for *C. albicans* wild type and *gpr*1Δ *gpa*2Δ cells from each gut compartment: BF1, bright field 1; CH2, AF488 (β-glucan exposure); CH6, side scatter; CH7, Hoechst 33,342 (DNA); BF2, bright field 2; CH10, ConA-AF647 (mannan exposure). For all experiments, means and standard deviations are from three independent experiments (or n = 3 mice), and statistical analyses were performed using the Mann-Whitney *U* test using Prism 5: * p ≤ 0.05.

Lactate-induced β-glucan masking is dependent on the G-protein coupled receptor-like protein, Gpr1, and its G-protein partner, Gpa2 (Ballou et al., 2016). Gpr1 is the closest *C. albicans* homologue to human GPR81 (Ballou et al., 2016), which stimulates lipolysis in a lactate-dependent fashion. The gut fermentation acids we tested possess common structural features to lactate, most obviously around their carboxyl group (Fig. 3A), suggesting that some SCFAs may influence β-glucan exposure by acting as Gpr1 agonists or antagonists. Therefore, we tested whether, like lactate, the effects of acetate and butyrate upon β-glucan exposure are dependent on Gpr1 and Gpa2. As expected (Ballou et al., 2016), lactate-induced β-glucan masking was blocked in a *gpr*1Δ *gpa*2Δ double mutant (Fig. 3B). Once again, acetate and butyrate promoted β-glucan exposure in wild type cells. Interestingly, in both cases this effect was attenuated in *gpr*1Δ *gpa*2Δ cells, although for acetate this attenuation was not statistically significant (Fig. 3B). This was consistent with the hypothesis that butyrate, and possibly acetate, act as antagonists of Gpr1/Gpa2-mediated β-glucan masking.

We exploited this observation to examine whether Gpr1/Gpa2-mediated β-glucan masking affects fungal burden and β-glucan exposure during gut colonisation in antibiotic-treated mice. Once again, fungal burdens were highest in the cecum compared to the small and large intestines, and the levels of β-glucan exposure were correspondingly lower in the cecum (Fig. 3C–E). Interestingly the large intestine was the only compartment in which significant differences in fungal burden and β-glucan exposure were observed between the *gpr*1Δ *gpa*2Δ mutant and its isogenic wild type control. In the large intestine, the levels of β-glucan exposure were significantly higher for *gpr*1Δ *gpa*2Δ cells, and the fungal burden was correspondingly lower. These data strongly support the inverse correlation between β-glucan exposure levels and *C. albicans* colonisation in the large intestine (colon), as reported previously (Sem et al., 2016). These observations also indicate that Gpr1 and Gpa2 promote β-glucan masking in the large intestine and that this contributes to colonisation of this gut compartment presumably by reducing immune recognition (Ballou et al., 2016).

### Differential β-glucan exposure between gut compartments is not dependent upon Wor1

The transcription factor Wor1 is known to be important for gut colonisation (Pande et al., 2013, Witchley et al., 2019). Therefore, as a control, we tested whether Wor1 inactivation compromises the ability of *C. albicans* to tolerate individual gut bacterial fermentation acids by examining growth in the presence of acetate, lactate, propionate or butyrate *in vitro*. No obvious differences between *wor*1Δ and wild type cells were detected (Supplementary Fig. 1).

**Fig. 4:**
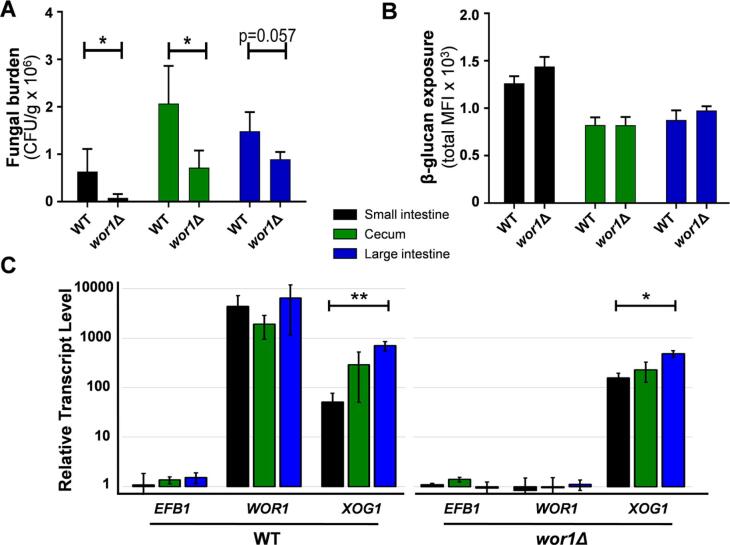
Impact of Wor1 on *C. albicans* β-glucan exposure and *XOG1* expression levels in the gut. (A) Mice (n = 3) were colonised with *C. albicans* wild type (SC5314) or *wor*1Δ cells (CAY189), sacrificed after four days, and fungal burdens (CFUs) quantified in the small intestine, cecum and large intestine. (B) β-glucan exposure on *C. albicans* cells from the same gut compartments by imaging flow cytometry (Fig. 1). Means and standard deviations are from n = 3 mice, and statistical analyses were performed using the Mann-Whitney *U* test using Prism 5: * p ≤ 0.05. (C) RNA was prepared from the contents of the small intestine, cecum and large intestine, and from control *C. albicans* cells grown at 30 °C in GYNB. The levels of each target mRNA in these preparations were quantified by qRT-PCR relative to the internal *ACT1* mRNA control, and then normalised against the transcript level in the control *C. albicans* cells grown *in vitro*. Means and standard deviations are from three independent experiments. The data were analysed statistically using one-way analysis of variance (ANOVA) using Prism 5: * p ≤ 0.05; ** p ≤ 0.01.

Wor1 targets include cell wall genes as well as transporter and metabolic genes (Zordan et al., 2007), and hence it was conceivable that Wor1 might influence β-glucan exposure within the murine gut. Therefore, we tested this. As expected, based on previous analyses of fungal loads in fecal samples (Pande et al., 2013), we observed lower fungal burdens for *C. albicans wor*1Δ cells in the small intestine, cecum and large intestine compared to control wild type cells (Fig. 4A). However, no difference was observed between wild type and *wor*1Δ cells with respect to their levels of β-glucan exposure in each of these gut compartments (Fig. 4B), suggesting that Wor1 is not required for β-glucan masking in the large intestine.

*XOG1* encodes a secreted exoglucanase that shaves exposed β-glucan from the *C. albicans* cell surface in response to lactate (Childers et al., 2020). Therefore, to further examine a potential role for Wor1 in β-glucan masking, we tested whether Wor1 regulates *XOG1* gene expression in the gut. *XOG1* mRNA levels were measured by qRT-PCR in *C. albicans* cells isolated from the small intestine, cecum and large intestine. *XOG1* mRNA levels were normalised against the *ACT1* mRNA internal control in these compartments, and then expressed relative to *XOG1* mRNA levels under control conditions (growth in GYNB at 30 °C). The *WOR1* and *EFB1* mRNAs were also included as controls (*EFB1* encodes the translation elongation factor EF-1β). Compared to the control GYNB-grown *C. albicans* cells, the *EFB1* mRNA was expressed at roughly similar levels in fungal cells isolated from the small intestine, cecum and large intestine (Fig. 4C). In contrast, the *WOR1* mRNA was expressed at much higher levels in wild type *C. albicans* cells from the gut compartments compared to the *in vitro* grown control cells. As expected, negligible levels of *WOR1* mRNA were detected in *wor*1Δ cells (Fig. 4C). The levels of both the *EFB1* and *WOR1* mRNAs did not differ significantly between compartments. However, *XOG1* mRNA levels were about 10-fold higher in wild type *C. albicans* cells from the cecum and large intestine compared to cells from the small intestine (Fig. 4C). This correlated with our observation that *C. albicans* cells from the cecum and large intestine display lower levels of β-glucan exposure than cells from the small intestine (Fig. 1B, 2B and 3B). The inactivation of *WOR1* did not dramatically affect the *XOG1* mRNA levels in any of these gut compartments (Fig. 4C), suggesting that Wor1 does not strongly influence *XOG1* expression. These data suggest that β-glucan masking acts in parallel with Wor1-dependent factors to promote gut colonisation.

## A mannan grazing gut bacterium promotes β-glucan exposure on *C. Albicans* cells

Our data suggested that Gpr1 and Gpa2 promote β-glucan masking in the large intestine, but that other factors are at play. Certain colonic bacteria, such as *Bacteroides thetaiotaomicron* and *Roseburia intestinalis,* are known to degrade and utilise *C. albicans* and *S. cerevisiae* mannans as a carbon source (Cuskin et al., 2015, La Rosa et al., 2019). The outer layer of the *C. albicans* cell wall is comprised of mannan fibrils (Hall and Gow, 2013, Lenardon et al., 2020), and this outer mannan layer normally masks the β-glucan buried in the inner layer of the cell wall (Graus et al., 2018, Hopke et al., 2018). Genetic, immunological or pharmacological perturbation of this outer mannan layer leads to increased β-glucan exposure (Hall and Gow, 2013, Wheeler and Fink, 2006, Wheeler et al., 2008, Galan-Diez et al., 2010, Hopke et al., 2016, Chen et al., 2019, Yang et al., 2022). Therefore, we reasoned that mannan-grazing bacteria in the gut might perturb the outer mannan layer of the *C. albicans* cell wall, leading to elevated β-glucan exposure at the fungal cell surface.

**Fig. 5:**
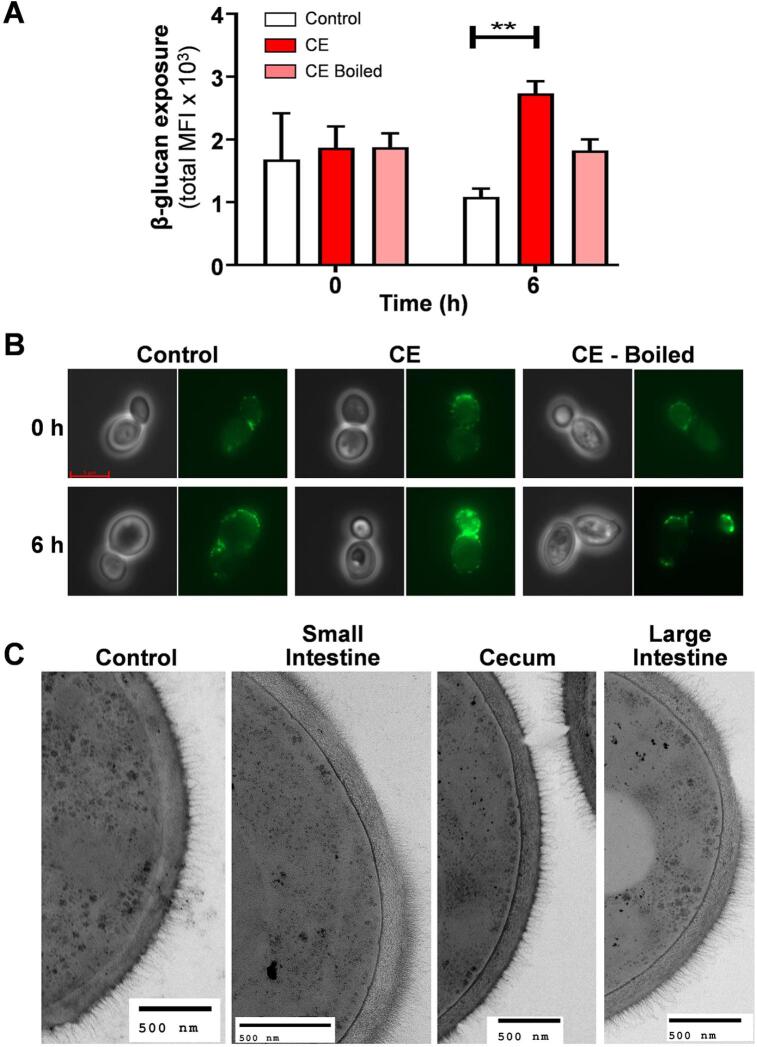
Effects of gut extracts upon β-glucan exposure and the *C. albicans* cell wall. (A) Antibiotic treated mice (n = 3) were sacrificed after four days, and soluble extracts prepared from their intestinal contents. A portion of these extracts was boiled to inactivate enzymes that might be present. In parallel, *C. albicans* SC5314 cells were pre-grown in GYNB at 30 °C, exposed to the colonic extracts, and the levels of β-glucan exposure on these cells at 0 and 6 h quantified by Fc-dectin-1 staining and imaging flow cytometry: Control untreated *C. albicans* cells, white; CE, cells treated with colonic extract, dark green; CE-boiled, cells treated with boiled colonic extract, pale green. Means and standard deviations are from three independent experiments, and statistical analyses were performed using the Mann-Whitney *U* test using Prism 5: ** p ≤ 0.01. (B) Images of representative cells from these assays by DIC and fluorescence microscopy. (C) Transmission electron microscopy (TEM) images of the cell walls of *C. albicans* cells incubated for 6 h with soluble extracts from the small intestine, cecum or large intestine of mice, and control *C. albicans* cells grown *in vitro* in GYNB at 30 °C. (For interpretation of the references to colour in this figure legend, the reader is referred to the web version of this article.). (For interpretation of the references to colour in this figure legend, the reader is referred to the web version of this article.)

To test this, we first prepared soluble colonic extracts from uninfected mice. Briefly, gut compartments were removed from the mice, the contents macerated in buffer and centrifuged to remove particulate matter, and the supernatants taken for analysis. A portion of each extract was then boiled to inactivate enzymes that were present. Wild type *C. albicans* SC5314 cells were then exposed to these colonic extracts, boiled extracts or a PBS control for zero or six hours, and levels of β-glucan exposure then quantified by Fc-dectin-1 straining and flow cytometry. Treatment with the colonic extract led to elevated β-glucan exposure levels compared to cells treated with the PBS or boiled extract controls (Fig. 5A, B). We then examined *C. albicans* cells from gut compartments by transmission electron microscopy (TEM). Unlike *in vitro* grown control cells, the cell walls of *C. albicans* cells from the small intestine, cecum and large intestine displayed noticeable “divots” in their outer mannan layer (Fig. 5C), consistent with the action of mannan grazing bacteria on the *C. albicans* cell surface.

**Fig. 6:**
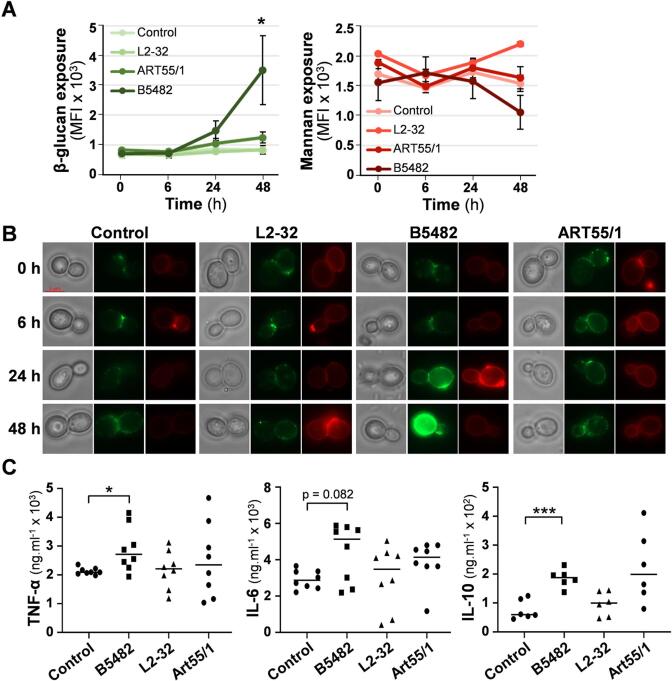
Effects of culture supernatants from gut anaerobes on β-glucan exposure by, and immunogenicity of, *C. albicans* cells. (A) *C. albicans* SC5314 cells were grown in GYNB, fixed in thimerosal, and incubated with M2GSC growth medium only (control) or with culture supernatants from *B. thetaiotaomicron* B5482, *B. adolescentis* L2-32 or *C. eutactus* ART55/1 grown in M2GSC. Cells were then stained with Fc-dectin-1 (for β-glucan) and Concanavalin A (for mannan), and the levels of exposure of both MAMPs quantified using a BD Fortessa flow cytometer. Means and standard deviations for the MFIs from three independent experiments are presented, and the data analysed using the Mann-Whitney *U* test with Prism 5: * p ≤ 0.05; ** p ≤ 0.01. (B) Images of representative cells from these assays by DIC and fluorescence microscopy: β-glucan, green; mannan, red. (C) Fixed *C. albicans* cells from the 48 h timepoint were then incubated for 24 h with PBMCs (5 yeast to 1 PBMC) and TNF-α, IL-6 and IL-10 levels quantified. Each data point represents one sample of two from four different individuals. Means are presented, and the statistical analysis were performed using ANOVA with the Bonferroni post-hoc test: *, *p* ≤ 0.05; **, *p* ≤ 0.01; ***, *p* ≤ 0.001. (For interpretation of the references to colour in this figure legend, the reader is referred to the web version of this article.). (For interpretation of the references to colour in this figure legend, the reader is referred to the web version of this article.)

We then tested the effects of co-incubating *C. albicans* SC5314 cells with culture supernatants from the fungal-mannan-degrading gut bacterium *B. thetaiotaomicron* B5482. We quantified the degree of β-glucan and mannan exposure on the fungal cell surface by cytometry of *C. albicans* cells co-stained with Fc-dectin-1 (for β-glucan) and Concanavalin A (for mannan). As controls we also examined the effects of culture supernatants from gut bacteria that degrade starch (*Bifidobacterium adolescentis* L2-32 (Belenguer et al. 2006)) or plant β-glucan (*Coprococcus eutactus* ART55/1 (Alessi et al., 2020)). No significant changes in β-glucan or mannan exposure were observed over a 24 h period for the *B. adolescentis* L2-32 and *C. eutactus* ART55/1 controls (Fig. 6A, B). However, co-incubation with supernatants from the fungal mannan degrader *B. thetaiotaomicron* B5482 led to increased β-glucan exposure, and decreased mannan exposure (Fig. 6A-B). Furthermore, these changes correlated with significantly enhanced release of the cytokines TNFα, IL-6 and IL-10 from human polymorphonuclear leukocytes exposed to *C. albicans* cells treated with the *B. adolescentis* supernatants, relative to the untreated control (Fig. 6C). This indicates that cross-kingdom interactions between mannan-grazing gut bacteria and *C. albicans* can influence immune responses against this fungus.

## Discussion

The recognition of β-glucan plays a major role in antifungal immunity both in mice and humans (Dennehy and Brown, 2007, Ferwerda et al., 2009, Saeed et al., 2014, Drummond et al., 2016, Sem et al., 2016, Hardison and Brown, 2012). Also, an inverse correlation between β-glucan exposure and gut colonisation, based on a comparison of the fecal burdens in mice infected with different *C. albicans* cell wall mutants, has been reported (Sem et al., 2016). Therefore, we set out to test our working hypothesis that lactate-induced masking of β-glucan exposed at the *C. albicans* cell surface might promote colonisation of the murine gut. Some observations suggested that this scenario was unlikely. Firstly, additional host related inputs, such as ambient pH, hypoxia and iron limitation modulate the exposure of β-glucan at the *C. albicans* cell surface (Sherrington et al., 2017, Pradhan et al., 2019, Pradhan et al., 2018, Cottier et al., 2019). In mice, the pHs of the small intestine, cecum and large intestine are similar (about pH 5 (McConnell et al., 2008)), and iron is thought to be abundant in gut compartments (Miret et al., 2003), but steep oxygen gradients exist from the gut mucosae across the lumen (Albenberg et al., 2014, Zheng et al., 2015). Therefore, ambient pH and hypoxia, but not iron limitation, may have also contributed to the modulation of fungal β-glucan exposure under our experimental setup. Secondly, although a number of host receptors detect β-glucan (Erwig and Gow, 2016), dectin-1 does play a major role in the recognition of fungal β-glucan and, thereby, in antifungal immunity (Ferwerda et al., 2009, Hardison and Brown, 2012, Saeed et al., 2014, Drummond et al., 2016). Yet a comparison of dectin-1^-/-^ and wild type mice in an oral infection model revealed that dectin-1 is not needed to control *C. albicans* colonisation levels in the gut (Vautier et al., 2012). Thirdly, additional MAMPs contribute to the recognition of fungi by innate immune cells (Erwig and Gow, 2016). Fourthly, interactions between the fungus, host and microbiota in the gut are multifactorial and extremely complex (d'Enfert et al., 2021). These interactions include effects of microbiota-generated metabolites upon macrophages (Schulthess et al., 2019), mycobiota-induced IgA antibodies that influence fungal commensalism (Doron et al., 2021b), and effects of *C. albicans* upon systemic immune signalling pathways and responses (Shao et al., 2019, Doron et al., 2021a). Therefore, it seemed unlikely that lactate-induced β-glucan masking is a primary driver of gut colonisation by *C. albicans*.

Nevertheless, our data suggest that Gpr1/Gpa2-mediated β-glucan masking does significantly influence colonisation of the mouse large intestine. The inactivation of *GPR1* and *GPA2* did not appear to affect fungal burdens in the small intestine or cecum. However, *C. albicans gpr*1Δ *gpa*2Δ cells did display increased β-glucan exposure in the large intestine and a correspondingly reduced fungal burden in this compartment (Fig. 3C, D). In *C. albicans,* Gpr1/Gpa2 signalling mediates responses to a variety of molecules that are structurally related to lactate, such as butyrate, methionine and possibly acetate in addition to lactate itself (Fig. 3B) (Maidan et al., 2005, Ballou et al., 2016). Therefore, although Gpr1/Gpa2 signalling is likely to reflect the local concentrations of a family of molecules rather than lactate alone, Gpr1/Gpa2-mediated masking of β-glucan at the fungal cell surface does promote colonisation of the large intestine. It is worth noting that our experiments were performed in mice treated with antibiotics, which are likely to influence the levels of fermentation acids in the intestine. Consequently, *C. albicans* might display subtle differences in cell wall adaptation when the fungus exists as a commensal in the absence of antibiotics.

Wor1 plays a central role in promoting the gut commensalism of *C. albicans* (Pande et al., 2013, Noble et al., 2017). However, our data suggest that Wor1 does not significantly influence β-glucan exposure in the gut. *XOG1* encodes a secreted exoglucanase that shaves β-glucan from the *C. albicans* cell surface (Childers et al., 2020). *WOR1* inactivation did not affect *XOG1* mRNA levels or β-glucan exposure in gut compartments (Fig. 4C). This resonates with the observation that Wor1 primarily regulates metabolic genes that promote the assimilation of carbon sources associated with the intestine, such as fatty acids and *N*-acetylglucosamine (Pande et al., 2013). Indeed, we observed that Wor1 inactivation influenced fungal burdens in all three gut compartments, whereas *gpr*1Δ *gpa*2Δ cells only displayed significant differences in the large intestine (Fig. 3C and 4B). Therefore, both metabolic tuning and β-glucan masking correlate with gut colonisation, but the regulation of these processes in *C. albicans* appears to be largely independent.

Fungal colonisation of the gut is suppressed by the competing indigenous microbiome via colonisation resistance (Kennedy and Volz, 1985a, Kennedy and Volz, 1985b). The abundance of mannan grazing bacteria such as the obligate anaerobe *B. thetaiotaomicron* in distal gut compartments (Xu et al., 2003, Cuskin et al., 2015, Kastl et al., 2020, Liu et al., 2021), is likely to present a significant challenge to *C. albicans*. Our data suggest that exposure even to culture supernatants from such bacteria compromises the outer mannan layer of the *C. albicans* cell wall, leading to the exposure of β-glucan (Fig. 5, Fig. 6). Our data (Fig. 6) and other published studies (e.g. (Sem et al., 2016)) suggest that this would lead to enhanced immune responses against *C. albicans* cells and reduced levels of colonisation. Therefore, there is likely to have been evolutionary pressure*,* particularly in distal gut compartments, to develop active shaving of β-glucan by *C. albicans*, through a combination of exoglucanases (Xog1 (Childers et al., 2020)) and endoglucanases (Eng1 (Yang et al., 2022)) to enhance the fitness of the fungus in the context of cross-kingdom interactions and antifungal immunity.

In conclusion, our data indicate that, in response to fermentation acids generated by gut bacteria, the commensal fungus, *C. albicans*, reduces the exposure of the proinflammatory MAMP, β-glucan, at its cell surface to promote colonisation of the large intestine. This β-glucan masking appears to counteract the effects of the mannan grazing by gut anaerobes that disrupts the mannan outer layer of the *Candida* cell wall to reveal underlying β-glucan. These observations highlight the complexity of the cross-kingdom interactions between microbiota, fungus and host that influence *Candida* immunogenicity and colonisation (d'Enfert et al., 2021).

## Declaration of Competing Interest

The authors declare that they have no known competing financial interests or personal relationships that could have appeared to influence the work reported in this paper.
