# Supplementary material for: Impact of changes at the Candida albicans cell surface upon immunogenicity and colonisation in the gastrointestinal tract
Source: Cell Surf. 2022 Oct 17;8:100084. doi: 10.1016/j.tcsw.2022.100084 (PMC9589014; doi:10.1016/j.tcsw.2022.100084)
Supplement: Supplementary data 1 [file mmc1.pdf]

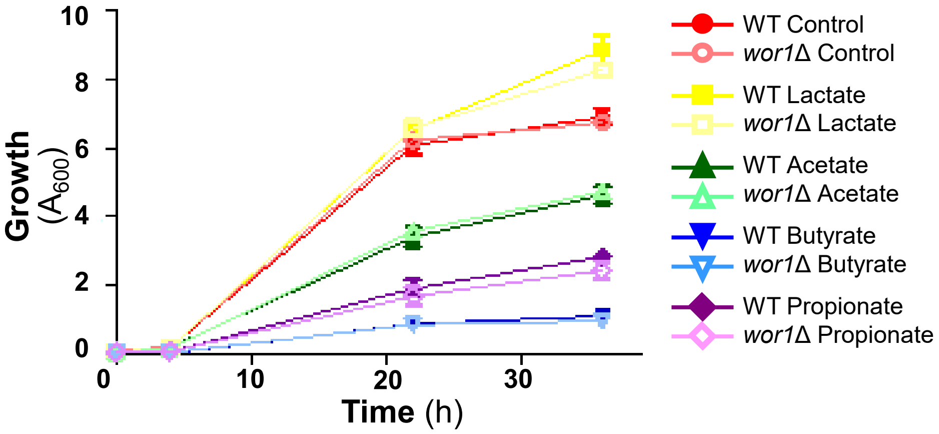

Supplementary Figure 1. Inactivation of *WOR1* does not affect growth in the presence of short chain fatty acids. The growth of *C. albicans* wild type (WT, CAY189) and *wor1* cells (CAY192) (Supplementary Table 1) was monitored at 30 °C in GYNB containing no SCFA (red), 55 mM lactate (yellow), 83 mM acetate (green), 56 mM butyrate (blue), or 67 mM propionate (purple).
