# Supplementary material for: Impact of changes at the Candida albicans cell surface upon immunogenicity and colonisation in the gastrointestinal tract
Source: Cell Surf. 2022 Oct 17;8:100084. doi: 10.1016/j.tcsw.2022.100084 (PMC9589014; doi:10.1016/j.tcsw.2022.100084)
Supplement: Supplementary data 2 [file mmc2.pdf]

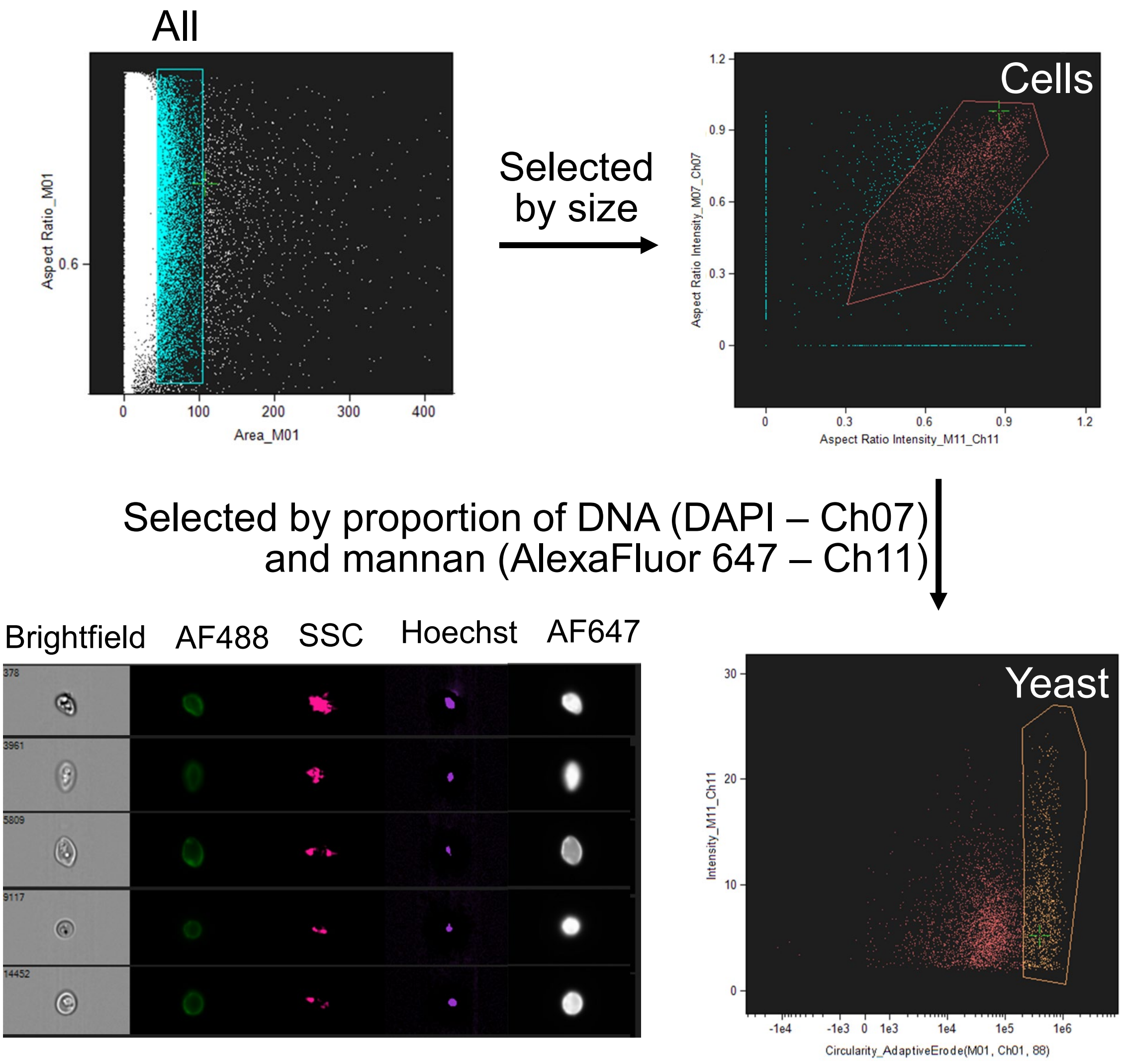

Supplementary Figure 2. Data cleaning strategy for ImageStream outputs. Initial screening based on aspect ratio intensity and area was performed to select potential *Candida albicans* cells, followed by proportion between mannan and DNA to remove artefacts and debris. Final gating was performed using circularity features based on image analysis.
