# Supplementary material for: Impact of changes at the Candida albicans cell surface upon immunogenicity and colonisation in the gastrointestinal tract
Source: Cell Surf. 2022 Oct 17;8:100084. doi: 10.1016/j.tcsw.2022.100084 (PMC9589014; doi:10.1016/j.tcsw.2022.100084)
Supplement: Supplementary data 3 [file mmc3.docx]

| **Table S1. Strains used in this study** | | | | | |
| --- | --- | --- | --- | --- | --- |
| **Strain** | **Name** | **Genotype** | **Source** |  |  |
| SC5314 | SC5314 | blood isolate | Gillum *et al.* (1984) |  |  |
| Ca372 | CAI4+CIp10 | *ura3∆::imm434/Δura3Δ::imm434, RPS1-CIp10 (URA3)* | Murad *et al.* (2000) |  |  |
| NM23 | *gpr1Δ* *gpa2Δ* | *ura3∆::imm434/Δura3Δ::imm434, gpa2Δ::hisG/gpa2Δ::hisG, gpr1Δ::hisG/gpr1Δ::hisG-URA3-hisG* | Maidan *et al.* (2005) |  |  |
| CAY189 | *wor1Δ* | *wor1::LEU2/wor1::HIS1* *MTLa/a* | Frazer *et al.* (2020) |  |  |
| CAY192 | *WOR1* wild type | Leu^+^ His^+^ *WOR1 MTLa/a* | Frazer *et al.* (2020) |  |  |

**References**

### Frazer C *et al.* (2020) Epigenetic cell fate in Candida albicans is controlled by transcription factor condensates acting at super-enhancer-like elements. *Nature Microbiol.* 5, 1374-1389.

Gillum AM *et al.* (1984) Isolation of the *Candida albicans* gene for orotidine-5'-phosphate decarboxylase by complementation of *S. cerevisiae* *ura3* and *E. coli pyrF* mutations. *Molec. Gen. Genet*. 198, 179-182.

Murad, AMA *et al.* (2000) CIp10, an efficient and convenient integrating vector for *Candida albicans*. *Yeast,* 16, 325-327

Maidan MM et al. (2005) The G protein-coupled receptor Gpr1 and the Galpha protein Gpa2 act through the cAMP-protein kinase A pathway to induce morphogenesis in Candida albicans. *Molec Biol Cell*. 16, 971-1986.
