# Supplementary material for: Impact of changes at the Candida albicans cell surface upon immunogenicity and colonisation in the gastrointestinal tract
Source: Cell Surf. 2022 Oct 17;8:100084. doi: 10.1016/j.tcsw.2022.100084 (PMC9589014; doi:10.1016/j.tcsw.2022.100084)
Supplement: Supplementary data 4 [file mmc4.docx]

**Table S2. Primers used in this study**

| **Name** | **Sequence** (5’🡪3’) | **Purpose** |
| --- | --- | --- |
| *ACT1* forward | ACCACCGGTATTGTTTTGGA | Quantification of the *ACT1* transcript |
| *ACT1* reverse | AGCGTAAATTGGAACAACGTG | “ |
| *EFB1* forward | AAGTCGAATCTATCAAGTCATTGAAC | Quantification of the *EFB1* transcript |
| *EFB1* reverse | GAGTGGCAGTAGTACCATCAATG | “ |
| *XOG1* forward | TGCTAAATGGTTGAATGGTGTC | Quantification of the *XOG1* transcript |
| *XOG1* reverse | GCATTATCGTAAGCACCCTCA | “ |
| *WOR1* forward | CAATTCAAATAGTTCTACATCAACCAA | Quantification of the *WOR1* transcript |
| *WOR1* reverse | TGCTTGTGGTGTTGGTACTGA | “ |
